# P-1831. *Clostridioides difficile* infection alters the prefrontal cortex transcriptomics in a preclinical model

**DOI:** 10.1093/ofid/ofae631.1994

**Published:** 2025-01-29

**Authors:** Deiziane Costa, Suemin E Yang, G Brett Moreau, Jae Hyun Shin, Cirle A Warren

**Affiliations:** University of Virginia, Charlottesville, Virginia; University of Virginia, Charlottesville, Virginia; University of Virginia, Charlottesville, Virginia; University of Virginia, Charlottesville, Virginia; University of Virginia, Charlottesville, Virginia

## Abstract

**Background:**

Dementia is associated with poor outcomes of *Clostridioides difficile* infection (CDI) in older adults. Changes in brain metabolites profile was detected in preclinical model of CDI. Recently, we discovered that CDI-promoted brain neuroinflammation occurs after peak of the infection. Here, we investigated how CDI changes the transcriptomics of prefrontal cortex of *C. difficile*-infected mice.

**Methods:**

6-month-old mice were infected with *C. difficile* and euthanized on day 7 post-infection (pi) and bulk RNA-sequencing was performed on prefrontal cortex. Changes in the main upregulated or downregulated genes were confirmed by protein assays. Spearman correlation analyses were also performed.

**Results:**

Infected mice developed diarrhea and weight loss, resulting in maximum weight loss and diarrhea on day 3 pi, which were resolved by day 7 pi. Differential gene expression analysis pointed 136 differentially expressed genes: 75 upregulated (*Lcn2, Plin4, Fkbp5, Slc38a5, Lrg1, Ucp2, Slc2a1, Spint1,* and *Trpv4*) and 61 downregulated (*Pprc1, Zfp169, Bnip5, Chrm4, Grm3, Hmgcr, Zfp804b, Gpm6b,* and *Ncam*) on day 7. Gene set enrichment (KEGG) analysis showed upregulation of genes associated with oxidative phosphorylation, Parkinson’s disease and Alzheimer’s disease, as well as downregulation of genes associated with axon guidance and neuroactive ligand-receptor interaction and T cell receptor signaling pathway in infected mice. Using protein assays, prefrontal cortex levels of GFAP, a marker of astrocytes, were increased in the infected mice, pointing to reactive astrocytes response. Increased levels of Lcn2, which can be secreted by astrocytes, followed by increased MPO and calprotectin in prefrontal cortex, cecum and serum of infected mice were detected. Levels of systemic MPO, a marker of inflammation, and fecal Lcn2, a marker of epithelial barrier disruption, correlates positively with the levels of Lnc2 in prefrontal cortex.

**Conclusion:**

Our findings demonstrate that CDI induces transcriptomic changes in the prefrontal cortex, including genes associated with oxidative phosphorylation and dementia secondary to Parkinson’s and Alzheimer’s disease, among others. Astrocytes-derived Lnc2 in the prefrontal cortex may contribute to the neuroinflammatory effect associated with CDI.

**Disclosures:**

**Jae Hyun Shin, MD**, Ferring: Grant/Research Support

